# A Visit to Foreign Dental Schools and Other Observations

**Published:** 1887-11

**Authors:** A. W. Harlan

**Affiliations:** Chicago, Ill.


ARTICLE VIII.

### A VISIT TO FOREIGN DENTAL SCHOOLS AND OTHER OBSERVATIONS.

BY A. W. HARLAN, M. D., D. D. S., CHICAGO, ILL.

France is so different from other foreign countries that a visitor usually spends most of his time in Paris. On previous occasions I had passed a few brief hours in other portions of the country, but most of my observations have been made during several visits to Paris, the home of many American dentists.

Away back in the "thirties" American dentists began practice in Paris, and now there are few cities in France with a population of one hundred thousand and upwards, where they are not to be found. Indeed, I believe there are several Americans in cities even smaller than that. I do not know the exact number, but it is my belief that there are from thirty to fifty English speaking dentists located in Paris. Of all those which I have visited, Paris seems to be more favored in this respect than any other continental city. I presume that many are drawn thither by the reports of the fabulous incomes of the few who have been long established in practice. For my own observations, I believe that many of the practices are over-estimated in the net incomes received, as there are few, if any, that yield as much as fifty thousand dollars per annum (250,000 francs).

When they take into account the cost of living in a foreign city—house rent, supplies, taxes, etc., entertainment of visitors, (like myself) and other expenses—probably there are not more than a dozen who can save above ten thousand dollars a year. The American dentist living in Paris is, of course, glad to see other Americans; but there are so many who visit Paris, that it must be very tiresome to see and entertain many who only come to visit, out of curiosity, those who are so located. Besides, they take up valuable time; and he must be very methodical indeed who can and will insist on being seen only after office hours. I was very glad to be taught such a lesson once by a prominent dentist in Paris, who sent word to me that he could only be seen before nine in the morning and after four in the afternoon, unless I had business with him. This I have not forgotten. We could profit by such a method at home, as it would enable us to accomplish much more in a day than can be done as things now are.

One Sunday morning I visited the Hospital and *Ecole Dentaire* of Paris, and was shown about the clinic rooms by M. Ch. Godon and Dr. Levett, the professor of operative dentistry. This school is the oldest in Paris, and it confers a diploma (D. E. D. P.) after a three years' course of study. At present the quarters at 23 Rue Richer are too small, and I was told that very soon they would remove into a more commodious building. The students were at work, occupying all the available chairs, using gold and other materials, rubber dam, etc. Everything was clean and orderly, and I was very favorably impressed with the workings during that morning. The students are required to spend some time in working with metals, ivory, steel, etc., that they may acquire dexterity in handling instruments. This is an important item in the education of a dentist, and much to be commended.

The candidate for a diploma must be twenty-one years of age, have attended three courses of lectures, deposited his specimen case, submitted to a preliminary examination, unless he be a doctor of medicine, an *officier de sante*, or have a diploma as midwife, when he or she can enter the second year's course of the school. The dental college is not a stock-holder's property, but was founded by subscriptions, and aided by the city of Paris. I was told that they had about 150,000 francs toward a permanent building (which fund has been increased), and this they will shortly occupy. There are a number of distinguished medical men and surgeons of Paris connected with the school, either as consultants, lecturers or professors. The school is in a flourishing conditlon, and had more than a hundred and twenty-five students last year. At present the diploma is not recognized by the State, as there is practically no law regulating dentistry in France. Any one may practice, I believe, by putting up a door-plate and announcing himself as a dentist. This is to be regretted, from the fact that medicine and surgery, and veterinary medicine also, are regulated by the Republic. In addition to the dental school and hospital, the Odontological Society of France meets in the college; also a dental benevolent society; and the faculty publish a journal—*L'Odontologie*—a monthly publication. This is a live journal, and I find something of interest in it every month. I have read it since its first issue, and I advise every one who reads French to do likewise, as it is only by comparison that we are able to finally judge of results. I wish the institution the support it justly deserves, and when again I visit France I hope to visit the new college building, and learn that the school has been endorsed by the State.

I was unable to visit the *Institut Odontotechnique*, which is the new school in Paris, but from a view of the outside I presume they have comfortable quarters; and a glance at the teaching corps will show that there are several well known names in the faculty, which is an assurance that they do good work. The new college publishes a journal also—the *Revue Odontologique*. *Le Progres Dentaire* and *L'Art Dentaire* are the only other dental journals of any consequence in Paris. My criticism of the dental journals would be that they contain too many translations of feeble efforts published in English or German, and too little original matter. Of late, however, they have been doing much better, and very soon I look for a still greater improvement on account of the increase of graduates from the dental schools.

France is the home of more original *brochures*, pam phlets, and works on dentistry, than any other country in the world; and the professional activity is only just being awakened. I was told that there are several well-known French dentists who have more calls on their time than they can possibly attend to, and that the incomes of some exceed 100,000 francs per annum, which I am sure must be so. Until recent years the bane of French operative dentistry has been the use of amalgams and cements, and the lack of thoroughness in filling roots. Indeed, many French dentists never think of filling a root when a tooth is to be replanted or a crown is adjusted; and many destroy the pulp and leave it in the tooth, fill the cavity and bore a vent-hole.

Too little time is spent in the preparation of cavities, and gold fillings, when inserted, are poorly done. These things will soon disappear as the new men enter the ranks and the old ones retire. The French are good artificers, and some of the most beautiful specimens of prosthetic dentistry that I have seen were made by French mechanical dentists. Too many French dentists use anæsthetics, and consequently many teeth are extracted that we at home would save. In the way of instruments and appliances they have as many as we, and all of ours too, to select from.

A thing that disgusts almost any dentist is to see the way in which some Americans, or people who call themselves such, mutilate the teeth of their countrymen or of any others who happen to fall into their clutches. What would we think of a dentist (?) at home who would solder a few teeth on a bar of platinum and then bore holes through living teeth, stick the ends of the bar through them, and cement this bridge with oxyphosphate, and call it permanent bridge-work? It is such operations as the above that bring reproach on American dentistry in Europe, as much as the bogus or unearned diplomas. The sooner they have a regulation of the practice of dental surgery in France, the better it will be for all concerned; then such Americans will be forced from practice, and all other foreigners, as well as natives. I have great faith in the sincerity of the French dentists, and believe that they will work out the problem of conservative dentistry if they only have the moral support and encouragement of their confreres in other countries. The members of the two principal societies are doing good work; and, while they do not quite agree with each other in the proposed manner of regulating the practice of dentistry in France, or the method of instruction of dental students, still the good results will follow. The discussions in the two societies, while too much taken up with extraneous matters, are beginning, from the reports which have reached me in the two official journals, to take a more elevated tone—are more serious, in fact, that they have ever been before.

The hospitality of the Frenchman is a thing which to me is incomparable. I had always been taught to believe that they were all selfish and without consideration for others: I found the reverse to be true. Almost a stranger, and little known, I was everywhere treated with the utmost cordiality, and friendships were formed which I am sure will endure forever, I hope at some time in the future (perhaps when there wlll be assembled an International Dental Congress) to reciprocate the many courtesies and acts of friendship heaped upon me by my confreres in France, and expect to welcome a number in Washington next September.

Americans are too prone to think that everything good or useful was invented or discovered at home; but if they would only read a little more, and travel with their eyes open, they would soon find that in many respects we have much to learn before perfection is attained, in art as well as in science; and a visit tc France would be not the least enjoyable journey that an American dentist could undertake.—*Independent Practitioner.*

